# Psychosocial Characteristics and Gestational Weight Change among Overweight, African American Pregnant Women

**DOI:** 10.1155/2012/878607

**Published:** 2012-11-21

**Authors:** Kelly C. Allison, Brian H. Wrotniak, Emmanuelle Paré, David B. Sarwer

**Affiliations:** ^1^Center for Weight and Eating Disorders, Department of Psychiatry, Perelman School of Medicine, University of Pennsylvania, Philadelphia, PA 19104, USA; ^2^Center for Clinical Epidemiology and Biostatistics, Perelman School of Medicine, University of Pennsylvania, Philadelphia, PA 19104, USA; ^3^Department of Physical Therapy, D'Youville College, Buffalo, NY 14201, USA; ^4^Division of Maternal-Fetal Medicine, University of Washington Medical Center, Seattle, WA 98195, USA

## Abstract

*Objectives*. To describe psychosocial factors identified as contributors of weight gain in the general population and to examine the relationship between these factors and gestational weight gain among low socioeconomic status, African American, overweight pregnant women. *Methods*. African American women (*n* = 120) with a pregravid body mass index ≥25 kg/m^2^ completed measures of eating, sleep, and depressed mood between 14 and 24 weeks of gestation. Weight was tracked. Descriptive statistics, correlations, and linear regression modeling were used to characterize the sample and examine predictors of gestational weight gain. *Results*. Four percent screened positive for night eating syndrome, with 32% consuming at least 25% of their daily caloric intake after dinner (evening hyperphagia). None met criteria for binge eating disorder; 4% reported occasional binge episodes. Cognitive restraint over eating was low. Participants slept 7.1 (SD = 1.9) h per night and reported 4.3 (SD = 3.6) awakenings per week; 18% reported some level of depressed mood. Night and binge eating were related to each other, sleep quality, and depressed mood. Eating due to cravings was the only psychosocial variable to predict gestational weight gain. *Conclusions*. Depressed mood, night eating, and nighttime awakenings were common in this cohort, while cognitive restraint over eating was low. Most psychosocial variables were not predictive of excess gestational weight gain.

## 1. Introduction

The impact of obesity and excess weight gain on pregnancy outcomes has become more salient in recent years, as the prevalence of overweight and obesity has grown [1]. In 2009-2010, 32% of women of childbearing age (20–39 yrs) were classified as obese and an additional 24% were overweight [1]. Rates of overweight and obesity among women of childbearing age also differ by ethnic group, with 51% of non-Hispanic white women and 74% of non-Hispanic black women between 20–39 years of age being overweight or obese. These high rates of obesity among childbearing women raise several important public health implications, including the increased risk of complications during pregnancy, such as gestational diabetes mellitus (GDM), cesarean delivery, and preeclampsia. Risks for the offspring include congenital malformations, stillbirth, macrosomia, shoulder dystocia, and neonatal intensive care stay [2–10].

Estimates across studies range from about 40% to 70% of women exceeding the 1990 IOM weight gain guidelines [11–14]; estimates with the 2009 guidelines [15] are few. Gestational weight gain for obese women above 6.8 kg (15 lb) substantially increases the risk of postpartum weight retention [16, 17], with a 0.18 kg (0.4 lb) increase above the baseline weight at 1 year postpartum for every pound gained [18]. Overweight and obese women are at a greater risk than their average-weight peers of retaining weight one year postpartum, thus, adding further to their excess adiposity [19]. In addition, there are racial disparities in gestational weight gain; African American women are more likely than their non-Hispanic white counterparts to retain weight postpartum [20, 21]. 

 Factors related to weight gain in the general population include behavioral and psychological influences such as disordered eating [22] sleep [23], and mood [24]. Despite the disparity in prevalence rates of obesity across racial groups, there have been a very few studies of minority populations identifying factors that may be related to excessive weight gain during pregnancy. However, it seems reasonable to suggest that the factors identified among the general population may also be related to excessive weight gain during pregnancy.

The forms of disordered eating most typically linked with obesity are binge eating disorder (BED), eating an objectively large amount of food accompanied by a loss of control on two or more days per week, and night eating syndrome (NES), a shift in the circadian pattern of eating characterized by consuming more than a quarter of energy intake after dinner and/or waking to eat at least twice per week [23, 25]. No studies have been published characterizing night eating behaviors during pregnancy, although given that sleep is more disrupted and that many women feel like they should “eat for two;” it seems plausible that night eating behaviors may be more common in pregnant women than in the general population. The prevalence of NES in the general population has been estimated at 1.5% [26] to 1.6% [27], and up to 7.5% [28] among obese women.

Some attention has been focused on the prevalence and influence of BED during pregnancy. Bulik and colleagues found that nearly 5% of women from the Norwegian Mother and Child Cohort Study (MoBa) met criteria for BED (using a modified criterion of at least one binge episode per week) at about 18-weeks gestation, with women of low socioeconomic status more likely to have BED [29]. This group further found that women with BED during pregnancy consumed more calories and fat and fewer key micronutrients than those without BED [30]. Thus, BED may be linked to weight-promoting behaviors during pregnancy.

Reduced sleep duration, which has been linked to obesity [31–34] and weight retention in the postpartum period [35] also may influence weight gain during pregnancy. Additionally, poor sleep quality early in pregnancy has been linked to increased depressed mood later in pregnancy [36, 37], and poor sleep quality in late pregnancy has been predictive of early postpartum depression [38]. Completing this intricate network of associations is the finding that postpartum depression acts as a predictor of postpartum weight retention [39]. However, descriptions of sleep during pregnancy are lacking among obese, minority populations. 

 Mixed evidence exists regarding the relationship between mood and gestational weight gain. In the general population, depressed mood has been associated with both increased and decreased appetite, and both are listed as symptoms of major depression [40]. Webb and colleagues reported that among a large cohort of women in the Pregnancy, Infection, and Nutrition (PIN) study, higher antenatal depressive symptoms were related to higher gestational weight gain [41]. 

 With the majority of women gaining more weight than that recommended by the IOM [16, 17], and with even stricter weight gain guidelines now in place for obese women [15], the purpose of the current study was to characterize the factors that have been linked to obesity in the general population among a cohort of African American, low socioeconomic status, overweight, and obese pregnant women seeking prenatal care and to test the relationship between these variables. The second purpose was to examine if these factors were linked to excessive gestational weight.

## 2. Materials and Methods

### 2.1. Participants

 Pregnant women who were attending an urban, university hospital-based obstetrical service for women on community-based health insurance were recruited. Two hundred eighteen women consecutively scheduled for prenatal care visits were screened for participation based on the following inclusion criteria: African American women 18 years of age or older with a pre-gravid (self-reported) body mass index (BMI) of ≥25 kg/m^2^, singleton pregnancy, no preexisting diabetes mellitus or autoimmune disorder, or regular use of steroid treatment. Seventy-five (34%) refused participation and 19 (9%) were ineligible due to exclusion criteria (11 were not African American, 4 were non-English speaking, 1 was pregnant with twins, and 3 had diabetes mellitus). After being consented, an additional 4 (2%) participants did not provide complete data at baseline, leaving 120 (55%) participants. 

### 2.2. Data Collection

 Women were recruited between 14 to 24 weeks of gestation. Eligible participants were identified before their scheduled clinic visits by clinic and study staff. Study staff approached eligible women and confirmed their age, gestational age, and pregravid BMI. If eligibility were confirmed, they participated in informed consent and were asked to complete a baseline survey packet as they waited for their appointments. 

 Medical chart abstractions were completed to calculate total pregnancy weight gain (last weight measured before birth—self-reported pregravid weight) and birth outcomes. Gestational weight gain data were available for 105 women (88%), as some women changed prenatal care providers or delivered elsewhere and no follow-up information was available. A raffle for a gift card was held each week for participants who had completed an assessment packet. 

### 2.3. Measures

 Participants first completed a demographic questionnaire that included information on medical and pregnancy histories and weight. To assess weight concern, we generated two items regarding pregravid and current pregnancy weight. These items were scored on a 0 (not at all concerned) to 4 (very concerned) scale.

**Disordered Eating and Eating Patterns**

To assess the eating, sleep, and mood patterns associated with NES, the Night Eating Questionnaire (NEQ) [42] was administered. The NEQ is a validated 14-item questionnaire with a range of 0–52 assessing the two core features of NES, evening hyperphagia and nocturnal ingestions, along with associated features, with higher scores indicating more severe pathology. A cut score of 30 is strongly suggestive of night eating syndrome [42].

To assess general disordered eating, we used the section of the Eating Disorders Examination-Questionnaire (EDE-Q) [43] that measures the frequency of objective overeating, binge eating (eating a large amount of food with a loss of control), and purging behaviors. These behaviors are the core symptoms associated with bulimia nervosa (BN) and BED. 

 We used item 51 of the Eating Inventory [44] to assess cognitive restraint over eating. This item reads, “On a scale of 1 to 6, where 1 means no restraint in eating, and 6 means total restraint, what number would you give yourself?”, with six specific descriptors following each rating. 

Eating habits that have been shown to affect weight gain, as measured in the Weight and Lifestyle Inventory (WALI) [45, 46], were included. Participants were asked to rate how much each impetus for eating had influenced their pregnancy weight gain on a 1 (does not contribute at all) to 5 (contributes the greatest amount) scale. Areas included were overeating at meals, snacking between meals, snacking after dinner, eating due to physical hunger, eating due to cravings, and eating when anxious, bored, stressed, angry, depressed/upset, or alone.

**Sleep**

Sleeping patterns were assessed using questions 1 through 6 of the Pittsburgh Sleep Quality Index (PSQI) [47]. These items assess sleep duration, sleep latency, sleep disturbances, and overall quality of sleep. The PSQI has been validated for pregnant populations, with good overall internal consistency [37, 48]. We also added questions assessing (1) how often they awoke and rose from bed in the past week, (2) the activities they engaged in during awakenings, and (3) the typical duration of each awakening.

**Mood**

Mood was assessed with the Edinburgh Postnatal Depression Scale (EPDS) [49]. This is a 10-item scale validated for both antenatal and postpartum use [49, 50], with scores ranging from 0 to 30. The validated antenatal cut score for “probable major depression” is 15 or greater, and a cut score of 13 or greater is used as a marker of “at least probable minor depression” [51].

### 2.4. Statistical Analysis

 Descriptive statistics, including frequencies, measures of central tendency, and standard deviations, were used to characterize demographic and survey data. Gestational weight gain was calculated using the last measured weight during pregnancy—self-reported pre-pregnancy weight. Participants were divided into groups according to whether they gained insufficiently, adequately, or in excess of the IOM guidelines. Correlations were used to test the associations between psychosocial variables and gestational weight gain. 

Multiple linear regression was used to examine the association between psychosocial variables and gestational weight gain. Maternal age, gestational age, pregravid BMI, and education were selected a priori as potential confounding variables that were forced into the model. Psychosocial variables associated with gestational weight gain (*P* < 0.1) were selected for inclusion in the model. A manual backwards stepwise procedure was then used to select the final model, and psychosocial variables with a significance level of <0.10 were retained as potential predictor variables. All analyses were conducted using SYSTAT 13.0 (Richmond, CA).

## 3. Results

One hundred twenty women, with a mean age of 25.2 (SD = 5.1) years and a mean pregravid BMI of 32.4 (SD = 7.8) kg/m^2^ completed the baseline questionnaire packet; 51% were obese and 49% were overweight before pregnancy. The majority had never been married and had a high school diploma or less (Table 1). Their employment status varied. Fourteen (11.7%) women had never been pregnant before the current pregnancy, 24 (20%) had previously been pregnant but had not given birth, and 82 (68.3%) were multiparous.

### 3.1. Eating Patterns

 The mean NEQ total score was 17.1 (SD = 5.9), with 4 (3.6%) women scoring ≥30, the clinical cut score for screening of NES. Examining the NEQ items that assess the two core features of NES, 38 (31.9%) of the participants reported consuming more than 25% of their daily caloric intake after dinnertime (NEQ no. 5), consistent with evening hyperphagia, and of these, 14 (11.8%) reported eating at least half of their intake after dinner. Seventy-seven (64.2%) reported awakening during the night more than once per week (nocturnal awakenings; NEQ #9). Eleven (9.4%) participants reported eating during the night at least half of the time they awoke (nocturnal ingestions; NEQ #12). Ten (8.5%) participants reported both evening hyperphagia (>25% of intake after dinner) and nocturnal ingestions (“about half the time” or more) on the NEQ.

No women reported binge eating at the clinical level of twice per week. Only 5 women reported an objective binge episode (i.e., eating a large amount of food with a loss of control). This included 1 participant reporting 4 binge episodes per month (the subclinical cut for BED), and an additional 4 reporting 1-2 episodes per month. Eighteen women (17.9%) reported having at least one objective overeating episode (i.e., eating a large amount of food without a loss of control) in the previous four weeks. Of these, 12 reported ≤3 episodes, two reported 4 episodes (about one per week), one reported 7 episodes, and 3 did not report a frequency. No women reported purging through vomiting, laxative use, or diuretics. Three women (2.6%) reported the use of vigorous exercise in response to eating. 

 The mean response to the cognitive restraint scale item was 2.7 (SD = 1.4), which corresponds to “often eat whatever I want, whenever I want.” The women were, on average, “a little” concerned regarding both their pregravid and current pregnancy weights (Table 1). Women did not heavily attribute their eating habits, such as snacking or eating in response to emotions, as contributing a considerable amount to their pregnancy weight gain. Items with the highest ratings were eating due to physical hunger, snacking between meals and after dinner, and eating in response to cravings (Table 2).

### 3.2. Sleep

 Participants reported sleeping an average of 7.1 (SD = 1.9) hours per night. They reported an initial sleep latency of 32.3 (SD = 38.6) minutes. Because of the large variance of this mean estimate, we also calculated the median sleep latency, which was 20 minutes. The mean number of awakenings when women rose from bed during the week was 4.3 (SD = 3.6), and they were awake for a mean of 40.6 (SD = 43.8) and a median of 25.0 minutes each time. Overall sleep quality was reported as “fairly good” at a mean of 2.8 (SD = 0.8; range very bad = 1 to very good = 4). Participants reported these activities while awake: 75.7% used the bathroom, 56.5% watched television, 7.0% read, 45.2% had a drink (beverage not specified), 26.1% ate, and 3.5% smoked. 

### 3.3. Mood

 The mean score on the Edinburgh Postnatal Depression Scale was 7.7 (SD = 6.0). Nine participants (7.9%) received a total score of 13 to 15, the subclinical cut for screening depressed mood in pregnancy, and an additional 12 women (10.5%) scored above 15, the clinical cut point. Thus, 21 (18.4%) women reported some level of depressed mood.

### 3.4. Correlations

 Data from all participants, including those without data for gestational weight gain (*n* = 15), were included in Table 3 to examine the cross-sectional relationships between the psychosocial measures; the *n* for each Pearson's *r* is provided. Gestational weight gain was not correlated with any of the disordered eating sleep, or mood factors, nor was it related to parity or smoking status. Pregravid BMI was also unrelated to most measures, showing only small, but significant relationships with cognitive restraint and depressed mood (Table 3). The NEQ produced stronger, positive associations with binge episodes, overall sleep quality, total hours of sleep, and depressed mood. Binge episodes were also significantly related to sleep quality and mood, but to a lesser extent. Women with more and better sleep reported less depressed mood. Pregravid BMI and the cognitive restraint item were not related to sleep quality or sleep duration. 

### 3.5. Gestational Weight Change

 According to the 2009 IOM weight gain guidelines for BMI group, 61 (58%) were considered excess gainers, 25 (24%) gained adequately, and 19 (18%) gained insufficiently; gestational weight gain could not be calculated for 15 participants. Gestational weight gain, examined both categorically and continuously, was not related to the method of delivery or oral glucose challenge test in the participants or birth weight or APGAR scores for the newborns.

As the only psychosocial variable correlated with gestational weight gain was “eating due to cravings” (see Tables 2 and 3), this variable was entered into a backward stepwise regression along with a priori control variables, including maternal age, gestational age, pregravid BMI, and education, to test a model of possible predictors of gestational weight gain. Eating due to cravings predicted weight at delivery adjusted *β* = 5.1 kg (11 lb) greater weight gain with every 1 unit more severe rating of eating due to cravings; 95% CI: 1.2 kg, 8.9 kg; *P* = 0.01. Adding parity to the model did not change these results, while adding smoking status attenuated the response slightly, adjusted *β* = 4.7 kg (10.4 lb) greater weight gain with every 1 unit more severe rating of eating due to cravings; 95% CI: 0.6 kg, 8.7 kg; *P* = 0.026. 

## 4. Discussion

 This is one of very few studies to describe eating patterns, sleep, mood, and gestational weight gain among a low-income, overweight and obese, African American cohort of pregnant women. The sample was characterized by the relatively common occurrence of evening hyperphagia and nocturnal ingestions, little evidence of binge eating, moderately disturbed sleep, and moderate levels of depressed mood. Just over the half of the sample gained excessively according to the 2009 IOM guidelines [15]. However, only eating due to cravings was significantly predictive of total gestational weight gain. 

 We found that only one participant endorsed a subclinical level of binge eating, but larger proportions reported some levels of objective overeating or subjective loss of control while eating in the previous month. Given that the women reported low levels of restraint and low levels of concern regarding their weight status, it is possible that they were not focused on limiting their intake. Instead, they may be evaluating large quantities not as a cause of concern, but as an acceptable practice during pregnancy. The low level of weight concern among this sample, both pregravid and during gestation, was not surprising, as Pike and colleagues found that African American women with BED describe lower levels of eating, weight, and shape concerns than white women [52]. Herring and colleagues also recently demonstrated that African American women show more concern over not gaining enough weight during pregnancy, with reinforcement for “eating for two” by family members, as opposed to concern for excess gain [53]. 

 The lack of active purging among this cohort supports this idea, as only a handful reported exercise in response to overeating, and no participants endorsed vomiting, laxative, or diuretic use. It seems unlikely that these women were engaging in “extreme” exercise characteristic of the compensatory behaviors seen in eating disorders. Bulik and colleagues' prevalence of 5% for subclinical or clinical BED among their Norwegian cohort [29] is higher than the 1% found in the current sample, although the sample size was significantly larger in the former study. Women in the Norwegian sample were more highly educated than the current sample, with 58% attending at least some college, and nearly all (97%) were married or cohabitating. This is an interesting contrast to the low level of subclinical cases of BED found in the current sample, suggesting that cultural and education differences may be influencing these behaviors. This theory contrasts reports that BED is as prevalent among African American women in the community as Caucasian women, but perhaps this dynamic is different in pregnancy [54]. 

Night eating behaviors were more common than binge episodes. Examining the two core criteria of NES separately [25], evening hyperphagia was present in a third of participants. As these were survey data, a more conservative estimate using “at least half of intake after dinner” still produced endorsement by 12% of the sample. The majority of women reported some frequency of nocturnal ingestions (56%), while 9% were eating at least half of the time they awoke. The high frequency of awakenings could be related directly to pregnancy, but these women were in their second trimester before significant discomfort and frequent nocturia would be present. Thus, these awakenings cannot be explained completely by discomfort due to pregnancy, suggesting that eating during the night was fairly common on a subclinical basis. To our knowledge, there are no population-based studies detailing the prevalence of these disorders in African American women during pregnancy to which we could compare these findings.

The women were sleeping about seven hours per night, with an awakening on most nights. Again, as this assessment occurred early in the second trimester, we would not expect to see large disturbances in sleep duration or quality generally, but we were more interested in latent sleep problems, such as insomnia or short sleep duration occurring independently of pregnancy, that might be related to weight gain. In the National Sleep Foundation 2010 Sleep Poll [55], African American respondents reported sleeping the least among all racial groups, averaging 6.2 hours per night during weekdays and 7.0 hours during weekends, with an additional hour in bed without sleeping. Thus, the current sample's sleep duration is similar to the normative amount for this racial group. The average initial sleep latency and the average time spent awake during an awakening were both highly variable in this sample, influenced by an outlier who estimated four-hour windows for both latency and awakenings. This issue suggests that it is difficult to characterize sleep patterns in this population neatly. 

 Nearly a fifth of the sample reported some level of depressed mood. The National Comorbidity Survey Replication [56] suggested that non-Hispanic black persons were 60% more likely than non-Hispanic white persons to have a lifetime history of depression, but rates of diagnoses in the previous 12 months were similar between groups. There have been no population studies comparing these racial groups' prevalence of depression during pregnancy, to our knowledge. However, the 18% identified in this sample is double the 9% rate estimated by Herring and colleagues using a cut score of 13 or greater in a sample in which 79% were Caucasian and 77% were college graduates [39]. However, perinatal samples of African American women (including both pregnant and postpartum) have found similar rates of depressed mood with the EPDS, with 19% scoring above 13 in one recent study [57]. Webb and colleagues found that depressed mood was positively related to gestational weight gain [41], suggesting that mood may be a significant target for intervention in a minority population to improve both mental health and gestational weight gain parameters. Despite this finding, mood was not a significant predictor of gestational weight gain in this sample. Unfortunately, we did not collect data on antidepressant use, so this may be a confounder in this relationship, one that may have impacted weight gain. 

 We confirmed in this sample that sleep quality is significantly correlated to mood. Further, disordered eating, both binge frequency and severity of night eating behaviors, was significantly linked to poor sleep quality and sleep latency, and for night eating, reduced sleep duration and depressed mood. This finding suggests that night-eating and binge-eating behaviors may be better predictors than BMI alone in identifying sleep problems and depressed mood during pregnancy. An intervention that focused on each of these domains would likely be useful, as poor sleep and depressed mood have each been linked to excess weight gain during pregnancy and postpartum weight retention, even if they were not significantly related in the current cohort [35, 39].

 Limitations to this study were its small sample size and the use of self-report data for pregravid weight. As this sample did not regularly attend gynecological appointments within the health system, it was not possible to record pregravid weights measured at recent visits for the majority of the sample. Additionally, we did not have psychosocial data in the third trimester of pregnancy.

Women in this obstetric clinic may also have had low literacy, given that almost a fifth of the sample did not have a high school diploma, which could have potentially influenced the validity of the data. Study staff members were trained to ask participants if they had questions or trouble understanding questions at various intervals while completing the packets, but this identified a small number of questions. Another limitation was the use of subscales or single items of validated surveys for some of the domains. Careful consideration was given to limit the size of the survey packet, given the time restrictions of the clinical visit. Based on the experience of the investigators, we selected the strongest and most salient surveys, subscales, and items. 

 In conclusion, several domains of psychosocial functioning were impaired among this African American, low-income cohort of overweight and obese pregnant women. Depressed mood was more common than previously reported in mainly white gestational cohorts, and sleep was disturbed, but not abnormally so compared to the general population. This study also identified that binge eating occurred at a very low frequency and night eating behaviors, including evening hyperphagia and nocturnal ingestions, were fairly common. However, only eating due to cravings significantly predicted excess gestational weight gain. Our difficulties in identifying modifiable psychosocial targets to include in potential interventions for controlling gestational weight gain may underlie the typically unsuccessful outcomes in interventions for controlling weight gain among overweight and obese women to date [18–20, 58–60]. Continued work is needed to identify modifiable targets to promote successful weight management in this population. 

## Figures and Tables

**Table 1:** Demographic information.

Demographic variables	*N* (percentages)
Marital status (*n* = 119)	92 (77%) never married
	21 (18%) married/live-in
	6 (5%) divorced/separated
Education (*n* = 119)	21 (18%) < HS grad
	51 (43%) HS or GED
	41 (34%) post-HS training/some college
	6 (5%) college graduate/postgraduate
Employment status (*n* = 118)	31 (26%) full time
	28 (24%) unemployed
	18 (15%) part time
	19 (16%) stay-home mother
	16 (14%) student
	6 (5%) unable to work

	Mean (SD)

Pregravid body mass index (*N* = 119)	32.4 (7.8) kg/m^2^
Weight concern for pregravid weight (*n* = 116)	1.7 (1.5)
Weight concern during pregnancy (*n* = 115)	1.8 (1.5)

	Median

Number of previous pregnancies (*n* = 118)	2
Number of previous live births (*n* = 118)	1

Due to rounding error, some percentages do not add to 100%.

**Table 2:** Attributions for the influence of eating habits on weight gain during pregnancy and their Pearson correlations with gestational weight gain.

Eating behavior	Mean (SD)	Correlation (*r*) with GWG
Overeating at meals	1.8 (1.1)	.03
Snacking between meals	2.3 (1.0)	.16
Snacking after dinner	2.2 (1.1)	.15
Eating because I feel physically hungry	2.4 (1.3)	.09
Eating because I crave certain foods	2.1 (1.1)	.21*
Eating when anxious	1.5 (1.0)	.07
Eating when tired	1.4 (0.9)	−.12
Eating when bored	1.4 (1.0)	−.02
Eating when stressed	1.6 (1.1)	.08
Eating when angry	1.6 (1.2)	−.08
Eating when depressed/upset	1.6 (1.1)	.07
Eating when alone	1.6 (1.1)	−.02

Items are extracted from the eating habits section of the weight and lifestyle inventory [45], and each is rated on a scale of 1 (does not contribute at all to weight gain) to 5 (contributes the greatest amount). GWG is gestational weight gain. *P < 0.05.

**Table 3:** Pearson correlations (*r*) between gestational weight gain, pregravid BMI, disordered eating, sleep, and mood variables.

Correlation *n *	Gest. weight gain	Pregravid BMI	NEQ total	Restraint scale item	No. of overeating episodes past 4 weeks	No. of binge episodes with loss of control	Minutes to fall asleep	Hours of sleep	Sleep quality	EPDS total	Parity	Smoking status
Gestational weight gain	1.00 105											
Pregravid BMI	−0.025 105	1.000 119										
NEQ total	0.053 96	−0.040 109	1.000 110									
Restraint scale item	−0.023 83	0.221* 95	−0.033 89	1.000 95								
No. of overeating episodes past 4 weeks	0.057 95	−0.021 107	0.184 99	0.085 86	1.000 107							
No. of binge episodes with loss of control	0.104 96	0.008 108	0.343** 101	−0.020 86	0.655** 105	1.000 109						
Minutes to fall asleep	−0.094 96	−0.038 110	0.252* 103	−0.013 89	0.244* 99	0.078 101	1.000 111					
Hours of sleep	0.055 97	0.114 110	−0.283** 104	−0.108 88	−0.177 100	−0.086 102	−0.362** 105	1.000 111				
Sleep quality	0.099 102	0.068 116	−0.428** 109	−0.130 94	−0.247* 106	−0.255** 108	−0.312** 110	0.515** 111	1.000 117			
EPDS total	0.039 100	−0.191* 113	0.523** 106	−0.010 92	0.255* 104	0.219* 105	0.326** 107	−0.234* 108	−0.361** 114	1.000 114		
Parity	−0.033 103	0.163 115	0.075 106	0.138 92	0.169 103	0.085 105	0.041 107	−0.065 107	−0.167 113	0.119 110	1.000 116	
Smoking status	−0.098 93	−0.094 105	0.144 97	0.138 85	0.287** 94	0.167 96	0.069 98	−0.160 98	−0.183 103	0.239* 100	0.263** 106	1.000 106

**Correlation is significant at the ≤0.01 level (2 tailed). *Correlation is significant at the ≤0.05 level (2 tailed).

Note: BMI is body mass index, NEQ total is the Night Eating Questionnaire total score, EPDS is the Edinburgh Postnatal Depression Scale. The restraint scale item is from the Eating Inventory. Sleep variables are subscales of the Pittsburgh Sleep Quality Index. Parity is the number of previous live births. Smoking status indicated whether they smoked or not during pregnancy. Higher scores on the NEQ, EPDS, and the subscales of the Pittsburgh Sleep Quality Index indicate more pathology. Higher scores on the restraint scale item indicate a higher cognitive restraint over eating.

## References

[1] Flegal KM, Carroll D, Kit BK, Ogden CL (2012). Prevalence of obesity and trends in the distribution of body mass index among US adults, 1999–2010. *The Journal of the American Medical Association*.

[2] Pasquali R, Pelusi C, Genghini S, Cacciari M, Gambineri A (2003). Obesity and reproductive disorders in women. *Human Reproduction Update*.

[3] Linné Y (2004). Effects of obesity on women’s reproduction and complications during pregnancy. *Obesity Reviews*.

[4] Baeten JM, Bukusi EA, Lambe M (2001). Pregnancy complications and outcomes among overweight and obese nulliparous women. *American Journal of Public Health*.

[5] Ray JG, Vermeulen MJ, Shapiro JL, Kenshole AB (2001). Maternal and neonatal outcomes in pregestational and gestational diabetes mellitus, and the influence of maternal obesity and weight gain: the DEPOSIT study. *QJM—Monthly Journal of the Association of Physicians*.

[6] Rosenberg TJ, Garbers S, Chavkin W, Chiasson MA (2003). Prepregnancy weight and adverse perinatal outcomes in an ethnically diverse population. *Obstetrics and Gynecology*.

[7] Weiss JL, Malone FD, Emig D (2004). Obesity, obstetric complications and cesarean delivery rate—a population-based screening study. *American Journal of Obstetrics and Gynecology*.

[8] Kristensen J, Vestergaard M, Wisborg K, Kesmodel U, Secher NJ (2005). Pre-pregnancy weight and the risk of stillbirth and neonatal death. *British Journal of Obstetrics and Gynaecology*.

[9] Cogswell ME, Perry GS, Schieve LA, Dietz WH (2001). Obesity in women of childbearing age: risks, prevention, and treatment. *Primary Care Update for Ob/Gyns*.

[10] Sarwer DB, Allison KC, Gibbons LM, Markowitz JT, Nelson DB (2006). Pregnancy and obesity: a review and agenda for future research. *Journal of Women’s Health*.

[11] Caulfield LE, Witter FR, Stoltzfus RJ (1996). Determinants of gestational weight gain outside the recommended ranges among black and white women. *Obstetrics and Gynecology*.

[12] Polley BA, Wing RR, Sims CJ (2002). Randomized controlled trial to prevent excessive weight gain in pregnant women. *International Journal of Obesity*.

[13] Olson CM, Strawderman MS, Reed RG (2004). Efficacy of an intervention to prevent excessive gestational weight gain. *American Journal of Obstetrics and Gynecology*.

[14] Kinnunen TI, Pasanen M, Aittasalo M (2007). Preventing excessive weight gain during pregnancy—a controlled trial in primary health care. *European Journal of Clinical Nutrition*.

[15] Institute of Medicine (2009). *Weight Gain During Pregnancy: Reexamining the Guidelines. Report of the Committee to Reexamine IOM Pregnancy Weight Guidelines*.

[16] Gore SA, Brown DM, West DS (2003). The role of postpartum weight retention in obesity among women: a review of the evidence. *Annals of Behavioral Medicine*.

[17] Institute of Medicine (1990). *Nutrition During Pregnancy, Weight Gain, and Nutrient Supplements. Report of the Subcommittee on Nutritional Status and Weight Gain During Pregnancy, Committee on Nutritional Status During Pregnancy and Lactation, Food and Nutrition Board*.

[18] Vesco KK, Dietz PM, Rizzo J (2009). Excessive gestational weight gain and postpartum weight retention among obese women. *Obstetrics and Gynecology*.

[19] Gunderson EP, Abrams B, Selvin S (2001). Does the pattern of postpartum weight change differ according to pregravid body size?. *International Journal of Obesity*.

[20] Crowell DT (1995). Weight change in the postpartum period: a review of the literature. *Journal of Nurse-Midwifery*.

[21] Siega-Riz AM, Viswanathan M, Moos MK (2009). A systematic review of outcomes of maternal weight gain according to the Institute of Medicine recommendations: birthweight, fetal growth, and postpartum weight retention. *American Journal of Obstetrics and Gynecology*.

[22] Allison KC, Stunkard AJ (2005). Obesity and eating disorders. *Psychiatric Clinics of North America*.

[23] Patel SR, Hu FB (2008). Short sleep duration and weight gain: a systematic review. *Obesity*.

[24] Stunkard AJ, Faith MS, Allison KC (2003). Depression and obesity. *Biological Psychiatry*.

[25] Allison KC, Lundgren JD, O’Reardon JP (2010). Proposed diagnostic criteria for night eating syndrome. *International Journal of Eating Disorders*.

[26] Rand CSW, Macgregor MD, Stunkard AJ (1997). The night eating syndrome in the general population and among post-operative obesity surgery patients. *International Journal of Eating Disorders*.

[27] Striegel-Moore RH, Franko DL, Thompson D, Affenito S, Kraemer HC (2006). Night eating: prevalence and demographic correlates. *Obesity*.

[28] Tholin S, Lindroos A, Tynelius P (2009). Prevalence of night eating in obese and nonobese twins. *Obesity*.

[29] Bulik CM, Von Holle A, Hamer R (2007). Patterns of remission, continuation and incidence of broadly defined eating disorders during early pregnancy in the Norwegian mother and child cohort study (MoBa). *Psychological Medicine*.

[30] Siega-Riz AM, Haugen M, Meltzer HM (2008). Nutrient and food group intakes of women with and without bulimia nervosa and binge eating disorder during pregnancy. *American Journal of Clinical Nutrition*.

[31] Roberts C, Troop N, Connan F, Treasure J, Campbell IC (2007). The effects of stress on body weight: Biological and psychological predictors of change in BMI. *Obesity*.

[32] Vorona RD, Winn MP, Babineau TW, Eng BP, Feldman HR, Ware JC (2005). Overweight and obese patients in a primary care population report less sleep than patients with a normal body mass index. *Archives of Internal Medicine*.

[33] Spiegel K, Tasali E, Penev P, van Cauter E (2004). Brief communication: sleep curtailment in healthy young men is associated with decreased leptin levels, elevated ghrelin levels, and increased hunger and appetite. *Annals of Internal Medicine*.

[34] Taheri S, Lin L, Austin D, Young T, Mignot E (2004). Short sleep duration is associated with reduced leptin, elevated ghrelin, and increased body mass index. *PLoS Medicine*.

[35] Gunderson EP, Rifas-Shiman SL, Oken E (2008). Association of fewer hours of sleep at 6 months postpartum with substantial weight retention at 1 year postpartum. *American Journal of Epidemiology*.

[36] Skouteris H, Germano C, Wertheim EH, Paxton SJ, Milgrom J (2008). Sleep quality and depression during pregnancy: a prospective study. *Journal of Sleep Research*.

[37] Skouteris H, Wertheim EH, Germano C, Paxton SJ, Milgrom J (2009). Assessing sleep during pregnancy. A study across two time points examining the Pittsburgh sleep quality index and associations with depressive symptoms. *Women’s Health Issues*.

[38] Wolfson AR, Crowley SJ, Anwer U, Bassett JL (2003). Changes in sleep patterns and depressive symptoms in first-time mothers: last trimester to 1-year postpartum. *Behavioral Sleep Medicine*.

[39] Herring SJ, Rich-Edwards JW, Oken E, Rifas-Shiman SL, Kleinman KP, Gillman MW (2008). Association of postpartum depression with weight retention 1 year after childbirth. *Obesity*.

[40] American Psychiatric Association (2000). *Diagnostic and Statistical Manual of Mental Disorders*.

[41] Webb JB, Siega-Riz AM, Dole N (2009). Psychosocial determinants of adequacy of gestational weight gain. *Obesity*.

[42] Allison KC, Lundgren JD, O’Reardon JP (2008). The night eating questionnaire (NEQ): psychometric properties of a measure of severity of the night eating Syndrome. *Eating Behaviors*.

[43] Fairburn CG, Beglin SJ (1994). Assessment of eating disorders: interview or self-report questionnaire?. *International Journal of Eating Disorders*.

[44] Stunkard AJ, Messick S (1985). The three-factor eating questionnaire to measure dietary restraint, disinhibition and hunger. *Journal of Psychosomatic Research*.

[45] Fabricatore AN, Wadden TA, Sarwer DB (2006). Self-reported eating behaviors of extremely obese persons seeking bariatric surgery: a factor analytic approach. *Obesity*.

[46] Wadden TA, Foster GD (2006). Weight and Lifestyle Inventory (WALI). *Obesity*.

[47] Buysse DJ, Reynolds CF, Monk TH, Berman SR, Kupfer DJ (1989). The Pittsburgh sleep quality index: a new instrument for psychiatric practice and research. *Psychiatry Research*.

[48] Jomeen J, Martin C (2007). Assessment and relationship of sleep quality to depression in early pregnancy. *Journal of Reproductive and Infant Psychology*.

[49] Cox JL, Holden JM, Sagovsky R (1987). Detection of postnatal depression: development of the 10-item Edinburgh postnatal depression scale. *British Journal of Psychiatry*.

[50] Murray D, Cox JL (1990). Screening for depression during pregnancy with the Edinburgh depression scale (EPDS). *Journal of Reproductive and Infant Psychology*.

[51] Matthey S, Henshaw C, Elliott S, Barnett B (2006). Variability in use of cut-off scores and formats on the Edinburgh postnatal depression scale—implications for clinical and research practice. *Archives of Women’s Mental Health*.

[52] Pike KM, Dohm FA, Striegel-Moore RH, Wilfley DE, Fairburn CG (2001). A comparison of black and white women with binge eating disorder. *American Journal of Psychiatry*.

[53] Herring SJ, Henry TQ, Klotz AA, Foster GD, Whitaker RC (2012). Perceptions of low-income African-American mothers about excessive gestational weightgain. *Maternal and Child Health Journal*.

[54] Smith DE, Marcus MD, Lewis CE, Fitzgibbon M, Schreiner P (1998). Prevalence of binge eating disorder, obesity, and depression in a biracial cohort of young adults. *Annals of Behavioral Medicine*.

[55] National Sleep Foundation (2010). *National Sleep Foundation 2010 Sleep in America Poll*.

[56] Kessler RC, Berglund P, Demler O (2003). The epidemiology of major depressive disorder: results from the national comorbidity survey replication (NCS-R). *The Journal of the American Medical Association*.

[57] King PAL (2012). Replicability of structural models of the Edinburgh postnatal depression scale (EPDS) in a community sample of postpartum African American women with low socioeconomic status. *Archives of Women’s Mental Health*.

[58] Asbee SM, Jenkins TR, Butler JR, White J, Elliot M, Rutledge A (2009). Preventing excessive weight gain during pregnancy through dietary and lifestyle counseling: a randomized controlled trial. *Obstetrics and Gynecology*.

[59] Guelinckx I, Devlieger R, Mullie P, Vansant G (2010). Effect of lifestyle intervention on dietary habits, physical activity, and gestational weight gain in obese pregnant women: a randomized controlled trial. *American Journal of Clinical Nutrition*.

[60] Phelan S, Phipps MG, Abrams B, Darroch F, Schaffner A, Wing RR (2011). Randomized trial of a behavioral intervention to prevent excessive gestational weight gain: the fit for delivery study. *American Journal of Clinical Nutrition*.

